# Intact LKB1 activity is required for survival of dormant ovarian cancer spheroids

**DOI:** 10.18632/oncotarget.4211

**Published:** 2015-06-05

**Authors:** Teresa Peart, Yudith Ramos Valdes, Rohann J. M. Correa, Elena Fazio, Monique Bertrand, Jacob McGee, Michel Préfontaine, Akira Sugimoto, Gabriel E. DiMattia, Trevor G. Shepherd

**Affiliations:** ^1^ Translational Ovarian Cancer Research Program, London Regional Cancer Program, London, Ontario, Canada; ^2^ Departments of Anatomy & Cell Biology, Schulich School of Medicine & Dentistry, The University of Western Ontario, London, Ontario, Canada; ^3^ Departments of Biochemistry, Schulich School of Medicine & Dentistry, The University of Western Ontario, London, Ontario, Canada; ^4^ Departments of Obstetrics & Gynaecology, Schulich School of Medicine & Dentistry, The University of Western Ontario, London, Ontario, Canada; ^5^ Departments of Oncology, Schulich School of Medicine & Dentistry, The University of Western Ontario, London, Ontario, Canada

**Keywords:** ovarian cancer, spheroid, LKB1, AMPK, tumour cell dormancy

## Abstract

Metastatic epithelial ovarian cancer (EOC) cells can form multicellular spheroids while in suspension and disperse directly throughout the peritoneum to seed secondary lesions. There is growing evidence that EOC spheroids are key mediators of metastasis, and they use specific intracellular signalling pathways to control cancer cell growth and metabolism for increased survival. Our laboratory discovered that AKT signalling is reduced during spheroid formation leading to cellular quiescence and autophagy, and these may be defining features of tumour cell dormancy. To further define the phenotype of EOC spheroids, we have initiated studies of the Liver kinase B1 (LKB1)-5′-AMP-activated protein kinase (AMPK) pathway as a master controller of the metabolic stress response. We demonstrate that activity of AMPK and its upstream kinase LKB1 are increased in quiescent EOC spheroids as compared with proliferating adherent EOC cells. We also show elevated AMPK activity in spheroids isolated directly from patient ascites. Functional studies reveal that treatment with the AMP mimetic AICAR or allosteric AMPK activator A-769662 led to a cytostatic response in proliferative adherent ovarian cancer cells, but they fail to elicit an effect in spheroids. Targeted knockdown of *STK11* by RNAi to reduce LKB1 expression led to reduced viability and increased sensitivity to carboplatin treatment in spheroids only, a phenomenon which was AMPK-independent. Thus, our results demonstrate a direct impact of altered LKB1-AMPK signalling function in EOC. In addition, this is the first evidence in cancer cells demonstrating a pro-survival function for LKB1, a kinase traditionally thought to act as a tumour suppressor.

## INTRODUCTION

Ovarian cancer is the most lethal gynecologic malignancy in the developed world, and the overall survival for women diagnosed with late-stage disease has remained largely unchanged for more than 25 years [1, 2]. Models that can be used to uncover the molecular events important for disease dissemination are crucial since the majority of women with ovarian cancer (over 75%) are diagnosed at advanced stage [3]. Intraperitoneal implants identified in these patients with advanced-stage disease are the result of single cells and multicellular aggregates, or spheroids, that adhere to the mesothelial lining of various abdominal organs to establish secondary lesions [4–6]. This is often accompanied by accumulation of ascites fluid within the peritoneal cavity, where cells in suspension are exposed to a unique set of microenvironmental cues, allowing this population of cells to form secondary metastases [7]. These non-adherent metastatic cells provide unique therapeutic challenges for treatment of ovarian cancer.

The biological significance and clinical relevance of multicellular spheroids has been documented in many different tumour types [8–14]. It is well accepted that spheroids more closely mimic the cell-cell, cell-matrix interactions, metabolic gradients, cellular viability and differentiation of malignant cells within a solid tumour than do conventional monolayer cultures [15]. We have shown that ascites-derived ovarian cancer cells in suspension form dormant multicellular aggregates characterized by quiescence and decreased Akt activity [16]. These dormant cells are subsequently able to re-enter the cell cycle and proliferate when they reach an adherent substratum. Ovarian cancer cells that are able to resist anoikis, and survive within peritoneal or ascitic fluid, have likely adapted key survival pathways to meet the nutrient and energy demands of this particular microenvironment.

A fundamental requirement of all cells is the ability to respond to various forms of metabolic stress and balance ATP consumption and generation. Under conditions where nutrients are low, AMP-activated protein kinase (AMPK) acts as a metabolic checkpoint by activating catabolic processes and inhibiting anabolic metabolism [17, 18]. It has been suggested that AMPK may function as a context-dependent tumour suppressor or oncogene [17]. Modest activation of AMPK may be cell protective, but prolonged or enhanced activation can be detrimental and result in growth arrest or cell death. AMPK is a heterotrimeric complex containing a catalytic α-subunit and two regulatory subunits, β and γ. When intracellular ATP levels are low, AMP or ADP directly bind to the γ regulatory subunits. This causes a conformation change in the complex that allows AMPK to be phosphorylated at threonine 172 on the α subunit [18]. The primary kinase responsible for phosphorylation at this site is Liver kinase B1 (LKB1) [19, 20]. LKB1 is encoded by *STK11*, which is commonly regarded as a tumour suppressor gene and is mutated in the rare hereditary autosomal dominant Peutz-Jeghers Syndrome. These patients experience benign intestinal hamartomatous polyps and have an increased risk of developing malignant tumours, including ovarian cancer [21]. In fact, recent studies have reported that loss of LKB1 expression may be an early event in high-grade serous ovarian cancer development [22, 23]. Despite this, solid evidence for *STK11* loss-of-function mutations has been identified in relatively few sporadic cancers.

Previous studies have shown that metabolic stress is induced when normal epithelial cells lose attachment to the extracellular matrix, resulting in a decreased ATP:ADP ratio and subsequent activation of AMPK [24, 25]. However, this suspension-induced AMPK activation has yet to be examined in tumour spheroids. In our study, we use a metastatic disease-relevant spheroid model to interrogate the function of the LKB1-AMPK pathway in ovarian cancer cells. Our results clearly demonstrate that LKB1 expression is maintained in nearly all ovarian cancer cells. Most importantly, we show that LKB1 and AMPK serve distinct functions in ovarian cancer cells and spheroids to regulate cell proliferation, cell survival and chemotherapy-resistance.

## RESULTS

### LKB1 and AMPKα expression and activity in ovarian tumours

**Figure 1:**
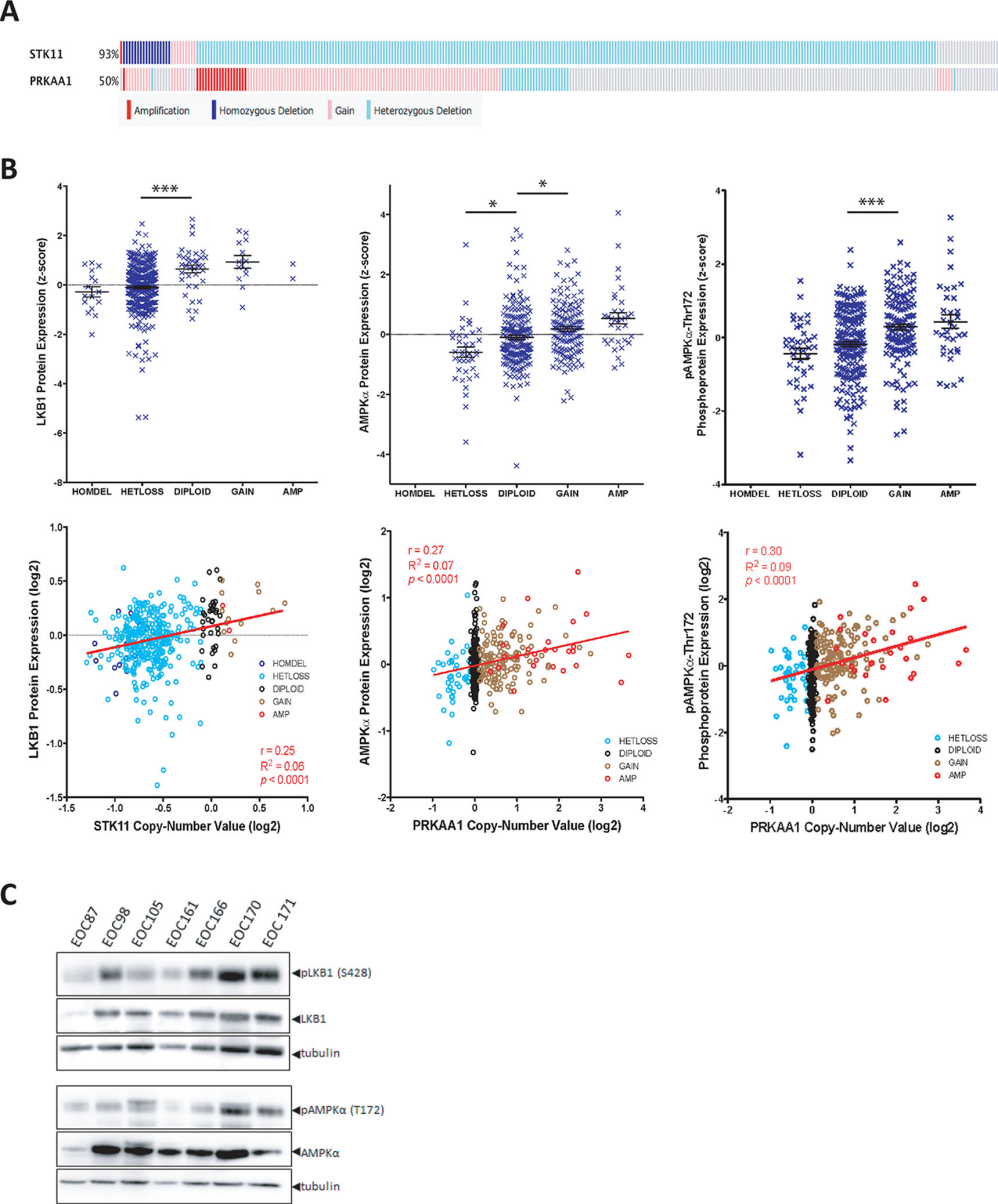
LKB1 and AMPKα expression in ovarian tumours **A.** Oncoprint analysis of copy number at the *STK11* and *PRKAA1* gene loci are depicted for 311 ovarian serous cystadenocarcinoma tumours obtained using the provisional TCGA dataset from cBioPortal. Amplification (red), copy number gain (pink), heterozygous deletion (light blue) and homozygous deletion (dark blue) are shown. **B.** Top panels: LKB1, AMPKα and phospho-AMPKα (Thr172) protein expression data from 397 serous ovarian tumours as determined by RPPA analysis and obtained from the TCGA dataset. Protein expression z-score is plotted against copy number. One-way ANOVA with Tukey's Multiple Comparison Test was performed (*, *p* < 0.05; ***, *p* < 0.001). Bottom panels: LKB1, AMPKα and p-AMPKα protein expression data was log_2_-transformed and plotted against log_2_-transformed gene copy number values. Pearson's r correlation, goodness-of-fit R^2^, and *p* values are reported. **C.** Lysates were generated from flash-frozen ovarian tumour samples from seven patients and immunoblot was performed to examine p-LKB1 (S428), LKB1, p-AMPK α (T172), and AMPKα expression in these samples.

Activity of the LKB1-AMPK signalling pathway is commonly thought to be tumour suppressive [26]. Multiple studies have suggested that single allelic inactivation of the *STK11* gene encoding LKB1 is sufficient to promote tumorigenesis, while other data suggests that biallelic loss may be required [27–30]. In order to examine the status of *STK11* (LKB1) and *PRKAA1* (AMPKα1) in serous ovarian tumours, we analyzed the gene copy number and reverse phase protein array (RPPA) data available from The Cancer Genome Atlas (TCGA) datasets using cBioPortal [31, 32]. The *STK11* gene exhibited copy-number alteration in 93% of 311 samples, with the majority (84%) comprising heterozygous deletion of the gene (Figure 1A). This single allelic loss correlated with decreased protein expression compared to samples with normal copy-number, and a positive correlation between *STK11* copy-number and LKB1 protein expression when we performed regression analysis on log2-transformed copy-number data (Figure 1B). When we examined LKB1 expression in ovarian tumour metastasis samples directly, however, we consistently observed detectable levels of phosphorylated and total LKB1 (Figure 1C). Therefore, despite single allele loss of *STK11*, LKB1 protein expression is maintained in metastatic ovarian cancer cells and may in fact serve an important function in late-stage disease.

AMPK has been described in many instances to serve as a tumour suppressor despite the lack of genetic evidence to demonstrate a loss of AMPK function in cancer [17]. Analysis of the *PRKAA1* gene (encoding AMPKα1) in TCGA data revealed copy-number alteration in 50% of serous ovarian tumours, with the majority (36%) comprising copy-number gain (Figure 1A). To determine whether *PRKAA1* copy-number correlated with protein expression, we plotted RPPA data against copy-number calls for both p-AMPKα (T172) and AMPKα. This demonstrated a significant increase in both phosphorylated and total AMPKα in samples with copy-number gain with a positive correlation between copy-number and AMPKα protein expression (Figure 1B). We also verified AMPKα expression and activity in lysates generated from ovarian tumour specimens directly (Figure 1C).

### Spheroids in patient ascites exhibit enhanced AMPK activity

**Figure 2:**
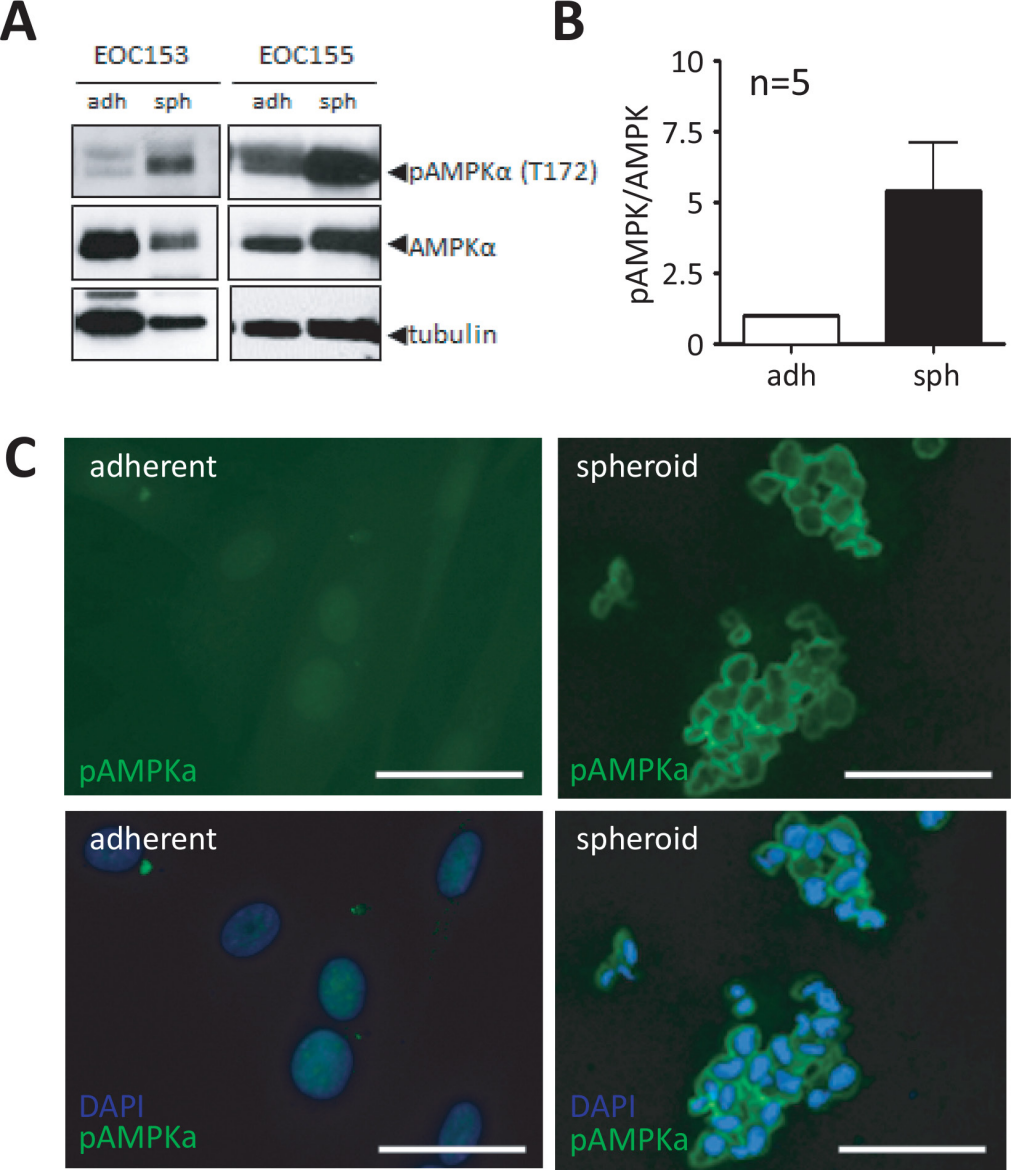
Native ascites spheroids have enhanced phosphorylated AMPK compared to adherent cells **A.** Representative immunoblot of p-AMPKα (Thr172) and total AMPKα in lysates isolated from native patient ascites-derived EOC spheroids as compared with passage-0 cultured cells. **B.** Densitometric analysis of immunoblot results from five independent patient ascites samples comparing p-AMPKα to total AMPKα. Data represents the mean ± SEM and Student's *t*-test (*, *p* < 0.05). **C.** Immunofluorescence analysis of p-AMPKα (green) in native ascites-derived spheroids analysis compared with matched primary adherent cells from the same patient (EOC 169). DAPI staining (blue) of nuclei is shown. Scale bar: 10 μm.

We have previously demonstrated that ovarian cancer cells form multicellular aggregates, or spheroids, and enter a dormant state, a process characterized by reduced proliferation and induced autophagy controlled in part by decreased AKT activity [16, 33]. Herein, we postulate that LKB1-AMPK signalling is another pathway mediating spheroid-induced dormancy due to its central function in responding to energy stress, such as nutrient deprivation and hypoxia [34], which are processes known to occur in these 3D structures [10, 15]. In order to evaluate this, we analyzed p-AMPK in spheroids filtered directly from patient ascites by western blot and immunofluorescence. Ascites spheroids from a number of different patient samples (*n* = 5) revealed a significant increase in p-AMPK in spheroids compared to matched adherent primary samples (passage-0) from the same patient (Figure 2A). Additionally, immunofluorescence revealed an intense signal for phosphorylated AMPKα (p-AMPKα) in the cytoplasm of native ascites-derived spheroids as compared with little to no detectable p-AMPK in matched adherent primary EOC cells (Figure 2B). These data indicate that AMPK activity is enhanced in malignant EOC cells in spheroids, which are the conduits of metastasis within ascites fluid.

### LKB1-AMPK signalling is activated during ovarian cancer spheroid formation

**Figure 3:**
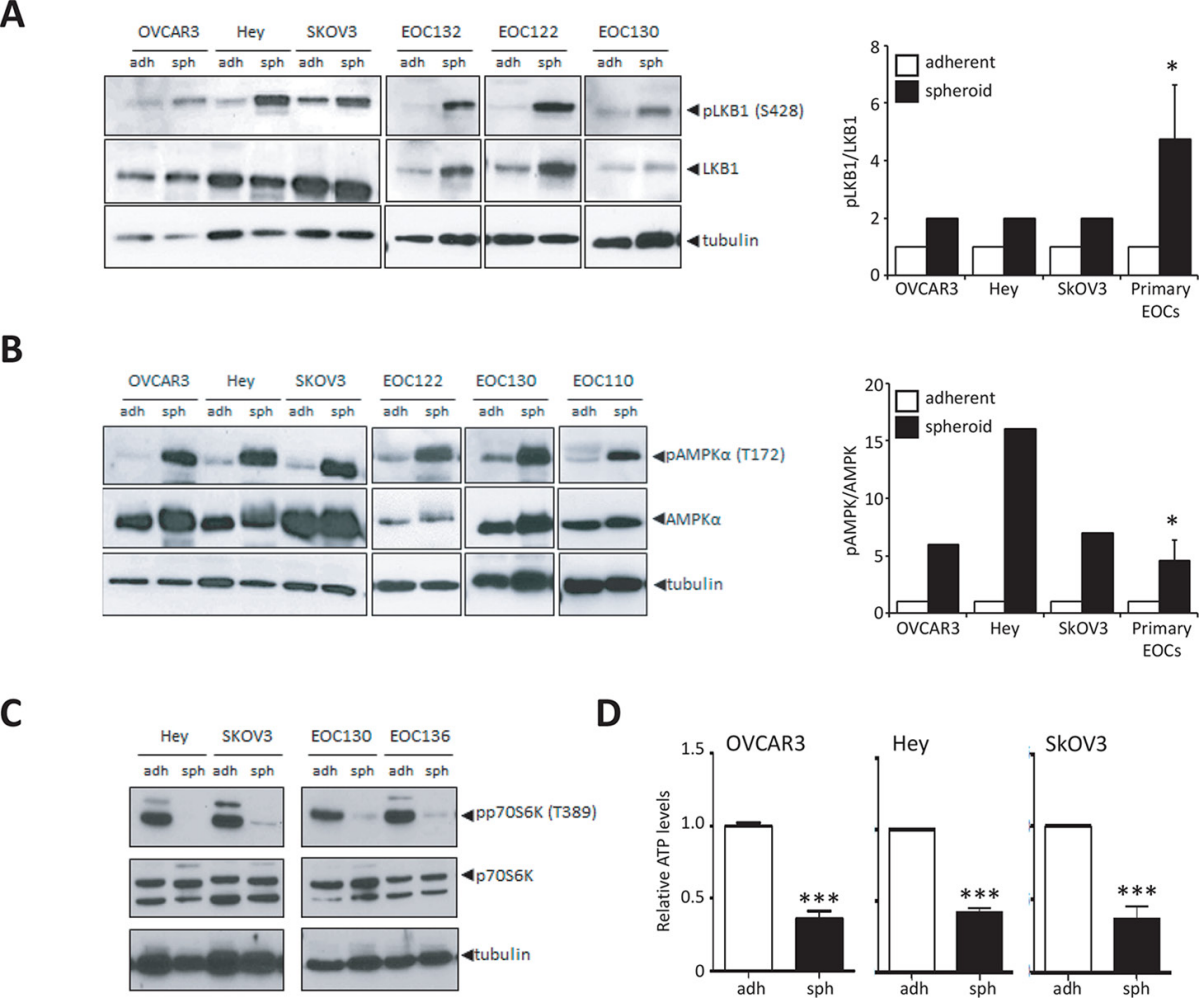
Increased LKB1-AMPK signalling activity in ovarian cancer spheroids **A.** Immunoblot analysis of OVCAR3, Hey and SkOV3 cell lines and ascites-derived EOC cells to determine levels of p-LKB1 (Ser428) and LKB1 expression in cultures of adherent cells (adh) and 3-day spheroids (sph). Densitometry was performed on immunoblot data from cell lines and ascites-derived EOC cells (*n* = 16) to quantify change in p-LKB1 expression relative to LKB1. Data is represented as mean ± SEM and Student's *t*-test for statistical significance (*, *p* < 0.05). **B.** Immunoblot analysis of OVCAR3, Hey and SkOV3 cell lines and ascites-derived EOC cells to determine levels of p-AMPKα (Thr172) and AMPKα expression in adherent cells (adh) and spheroids (sph). Densitometry was performed on immunoblot data from cell lines and ascites-derived EOC cells (*n* = 14) to quantify change in p-AMPKα expression relative to AMPKα. Data is represented as mean ± SEM and Student's *t*-test for statistical significance (*, *p* < 0.05). **C.** Immunoblot analysis of Hey and SkOV3 cell lines and ascites-derived EOC cells to determine levels of p-p70S6K (Thr389) and p70S6K as a measure of mTORC1 activity. **D.** Quantification of intracellular ATP levels in SkOV3, Hey and OVCAR3 cell lines using luminescence-based ATP assay CellTiter Glo^®^ cultured as adherent cells (adh) or spheroids (sph). Luminescence values were normalized to total protein in each sample. Data is represented as mean ± SEM and Student's *t*-test for statistical significance (***, *p* < 0.001).

Following our observation that AMPK activity is enhanced in native ascites spheroids, we sought to further investigate the regulation of this phenomenon using spheroids formed *in vitro*. The majority of ovarian cancer cell lines grown as adherent proliferating cells in culture exhibit low to undetectable levels of phosphorylated LKB1 (p-LKB1) (S428) and p-AMPK α (T172) (Figure 3A, 3B; Supplementary Figure S1). In contrast, western blot analysis revealed a significant increase in p-LKB1 and p-AMPK expression associated with spheroid formation when compared with adherent cells (Figure 3A, 3B). Although LKB1 phosphorylation does not affect its catalytic activity, phosphorylation at S428 has been shown to be important for the tumour suppressive functions of LKB1 [35, 36]. LKB1 can be localized to either the nucleus or cytoplasm, and it is the cytoplasmic pool of LKB1 that contributes to the tumour suppressive function of this kinase [26]. Thus, we performed cellular fractionation and immunoblotting to determine LKB1 localization and demonstrate that LKB1 is found in the cytoplasm in both adherent and spheroid cells (Supplementary Figure S2).

Since the LKB1-AMPK signalling pathway has been identified as a key negative regulator of mTORC1 signalling, we used this pathway as a downstream readout for the ability of LKB1-AMPK to rewire cellular metabolism in spheroids. Immunoblot performed on spheroids from cell lines and ascites-derived cells revealed a significant decrease in mTORC1 activity as determined by p70S6K1 phosphorylation (Figure 3C). This result implies that activation of LKB1-AMPK signalling in spheroids with the downstream inactivation of mTORC1 would lead to a significant decrease in cellular anabolic metabolism. To assess whether the overall energy metabolism of spheroid cells was also reduced, we determined levels of intracellular ATP in ovarian cancer cells in adherent and spheroid culture. Indeed, ATP levels were significantly lower in spheroids generated using three ovarian cancer cell lines compared to their adherent counterparts (Figure 3D). Taken together, our results demonstrate that LKB1-AMPK signalling appears to be intact and active in metastatic epithelial ovarian cancer, and that the overall metabolic state is altered in ovarian cancer spheroids with a concomitant response of activated LKB1-AMPK signalling.

### Forced AMPK activity in proliferating ovarian cancer cells induces cytostasis

**Figure 4:**
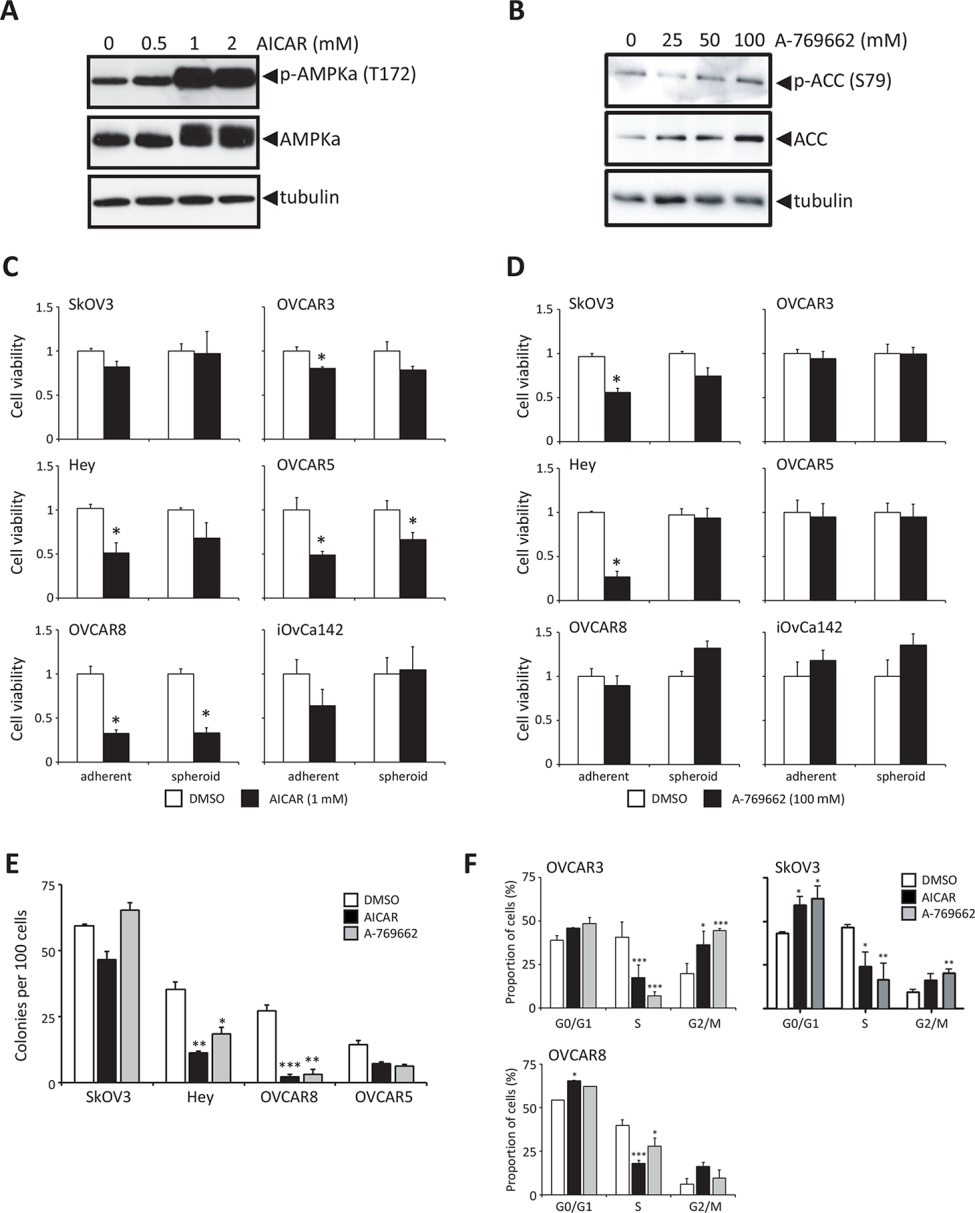
Stimulation of AMPK activity in proliferating ovarian cancer cells reduces growth and colony forming potential **A.** Immunoblot analysis of p-AMPKα (Thr172) and AMPKα expression in Hey cells treated for 24 h with 0.5, 1 or 2 mM AICAR. **B.** Immunoblot analysis of p-ACC (Ser79) and ACC expression in Hey cells treated for 24 h with 25, 50 or 100 μM A-769662. **C.** Cell viability was determined following 3 d of AICAR (1 mM) treatment (black bar), or DMSO control (white bar) of six ovarian cancer cell lines cultured as adherent cells and spheroids. Data is represented as mean ± SEM and Student's *t*-test for statistical significance (*, *p* < 0.05). **D.** Cell viability was determined following 3 d of A-769662 (100 μM) treatment (black bar), or DMSO control (white bar) of six ovarian cancer cell lines cultured as adherent cells and spheroids. Data is represented as mean ± SEM and Student's *t*-test for statistical significance (*, *p* < 0.05). **E.** Clonogenic assays were performed by seeding ovarian cancer cell lines (1, 000 cells per well of 6-well dish) treating with 1 mM AICAR, 100 μM A-769662, or DMSO control for 3 d, followed by growth recovery in complete media. Data is represented as mean ± SEM and one-way ANOVA with Tukey's Multiple Comparison test (*, *p* < 0.05; **, *p* < 0.01; ***, *p* < 0.001). **F.** Cell cycle analysis was performed by flow cytometry using BrdU and PI staining of OVCAR3, OVCAR8 and SkOV3 cells following 24 h of treatment with either 1 mM AICAR or 100 μM A-769662 (*n* = 2 for each cell line). Data is represented as proportion of cells in G0/G1, S and G2/M phases of the cell cycle. Data is represented as mean ± SEM and one-way ANOVA with Tukey's Multiple Comparison test (*, *p* < 0.05; **, *p* < 0.01; ***, *p* < 0.001).

To further investigate a potential causal relationship between active LKB1-AMPK signalling and the dormancy phenotype of ovarian cancer spheroids, we first sought to determine the effect of enforced LKB1-AMPK signalling on proliferating ovarian cancer cells. It has been previously demonstrated that treatment of ovarian cancer cells with AMP mimetic AICAR results in increased AMPK activity and decreased viability of adherent cells [37, 38]. Treatment of ovarian cancer cells with 1 mM AICAR led to a robust phosphorylation of AMPKα (Figure 4A). We also tested a more specific allosteric AMPK activator, A-769662, which stimulates AMPK directly without affecting the kinase domain [39]. Treatment of ovarian cancer cells with 100 μM of A-769662 resulted in activation of AMPK, indicated by increased phosphorylation of the downstream AMPK substrate acetyl-CoA carboxylase (ACC) (Figure 4B). We then assessed the effect of extended AMPK activation on the growth and viability of various ovarian cancer cell lines and ascites-derived cells. AICAR treatment decreased the viability of adherent cells (4/6 cell lines) after 3 d of treatment (Figure 4C), and this effect was not as robust in spheroids (2/6 cell lines). Treatment with A-769662 resulted in only a modest decrease in viable cell number in adherent-cultured cells (2/6 cell lines), and no effect on ovarian cancer spheroids (Figure 4D). We confirmed that both AICAR and A-769662 resulted in similar AMPK activation in treated adherent cells and spheroids (Supplementary Figure S3). The same differential effect between adherent cells and spheroids was observed by AICAR treatment of ascites-derived cells (Supplementary Figure S4A). When spheroids are allowed to reattach to tissue culture plastic, cells disperse based on the combination of cell proliferation and motility. AICAR treatment of spheroids generated from ascites-derived cells during reattachment led to a significant reduction in dispersion area (Supplementary Figure S4B). Since AICAR treatment of ovarian cancer cells in scratch assays exhibited no effect on cell motility over 24 hours (data not shown), this demonstrates further that forced pharmacologic AMPK activation is detrimental to proliferating ovarian cancer cells.

Following the observation that both AICAR and A-769662 reduce the proportion of viable ovarian cancer cells in proliferating adherent culture, we tested these agents on the clonogenic capacity of several ovarian cancer cell lines. Hey, OVCAR5 and OVCAR8 cells displayed reduced colony formation due to AICAR and A-769662 treatment (Figure 4E). Flow cytometry was performed to assess whether forced AMPK activation affected ovarian cancer cell cycle. Both compounds resulted in a decreased proportion of ovarian cancer cells in the S-phase of the cell cycle as early after 24 h of treatment, and in most instances with a respective increase in cells in G0/G1 and G2 (Figure 4F). We also assessed apoptosis in AICAR- and A-769662-treated ovarian cancer cells, and there was no effect of these agents on inducing programmed cell death to reduce viable cell number (data not shown). Taken together, these results suggest that forced AMPK activity in proliferating ovarian cancer cells acts to induce a cytostatic response, and may explain a function for the observed increase in endogenous p-AMPK in non-proliferating EOC spheroids.

### LKB1 is required for ovarian cancer cell survival and platinum resistance in spheroids

**Figure 5:**
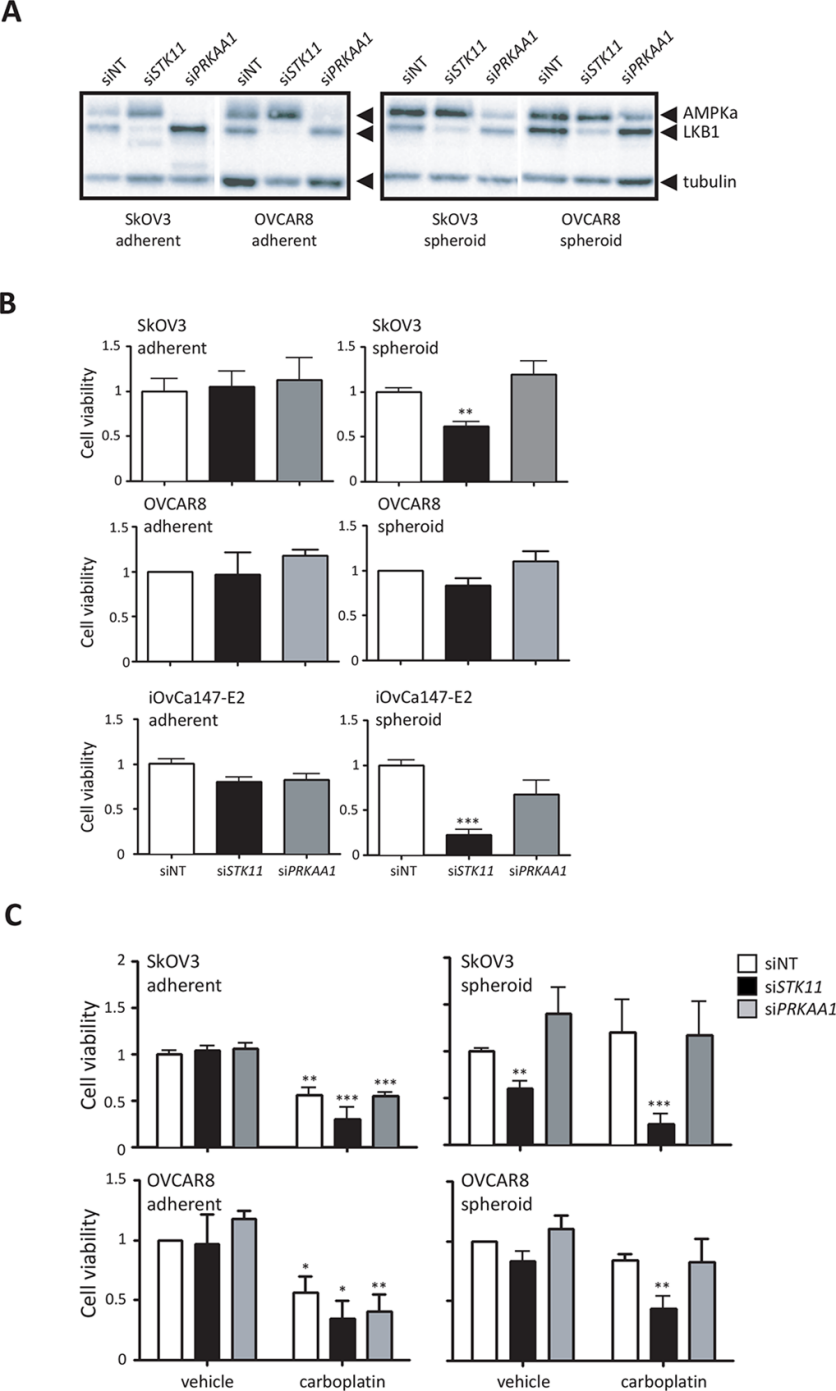
LKB1 expression is required to maintain cell viability and promote chemoresistance of ovarian cancer spheroids **A.** Immunoblot analysis of LKB1 and AMPKα expression in ovarian cancer cells transfected with *STK11* and *PRKAA1* siRNA pools, or control siRNA (siNT). Expression was determined 72 h after transfection in adherent cells, or 72 h after cells were seeded to form spheroids. **B.** Cell viability was determined after 72 h using CellTiter-Glo^®^ assay on siRNA-transfected SkOV3, OVCAR8 and iOvCa147-E2 cell lines cultured as adherent cells or spheroids. Data is represented as mean ± SEM and one-way ANOVA with Tukey's Multiple Comparison test (*, *p* < 0.05; ***, *p* < 0.001). **C.** The experiment was performed as in (B), but where SkOV3 and OVCAR8 cell lines were treated with 50 μM carboplatin at 72 h post-transfection in adherent culture or at the time of seeding to 24-well ULA cluster plate to form spheroids. Cell viability was determined after 72 h using CellTiter-Glo^®^ assay. Data is represented as mean ± SEM and one-way ANOVA with Tukey's Multiple Comparison test (*, *p* < 0.05; **, *p* < 0.01; ***, *p* < 0.001).

Given the relative insensitivity of ovarian cancer cells in spheroids to further activation of AMPK, we assessed the functional impact of attenuation of the LKB1/AMPK pathway in spheroids. We transfected SkOV3, OVCAR8 and iOvCa147-E2 ovarian cancer cell lines with pooled siRNAs against *PRKAA1* (AMPKα1) and *STK11* (LKB1). Effective knockdown of *STK11* and *PRKAA1* was achieved in both adherent monolayer cells and spheroids (Figure 5A). Cells in adherent culture were not sensitive to knockdown of either *PRKAA1* or *STK11* with respect to cell viability, most likely since these cells are proliferating and have low LKB1-AMPK signalling activity (Supplementary Figure S1). In marked contrast, loss of LKB1 expression significantly reduced cell viability in spheroids, whereas *PRKAA1* knockdown had little to no effect (Figure 5B). Performing analogous experiments in patient ascites-derived EOC cells, we observed a similar effect of reduced viability in spheroids due to *STK11* knockdown (Supplementary Figure S5).

Platinum-based chemotherapy is the standard for first-line treatment of metastatic epithelial ovarian cancer, yet the majority of patients will eventually recur with platinum-resistant disease [40]. Ovarian cancer spheroids are largely resistant to platinum treatment, likely due to their reduced proliferative state [16, 41]. To assess whether loss of LKB1-AMPK signalling can affect ovarian cancer spheroid sensitivity to platinum agents, adherent cell and spheroids transfected with *STK11* or *PRKAA1* siRNA were treated subsequently with carboplatin. We observed a dramatic reduction in cell viability in ovarian cancer spheroids when loss of LKB1 was combined with carboplatin treatment, whereas this effect was not seen is spheroids with *PRKAA1* knockdown, or in adherent cells under all treatment conditions (Figure 5C). These final results point to a critical role for LKB1 signalling in maintaining cell viability and achieving chemo-resistance in dormant ovarian cancer spheroids largely in an AMPK-independent manner.

## DISCUSSION

The distinct mode of metastatic spread whereby EOC cells transit the peritoneal cavity in suspension presents unique therapeutic challenges for treatment of advanced-stage ovarian cancer [42, 43]. Characterization of this unique population of non-adherent cells will provide insights into novel targets for treatment of this deadly disease. Our laboratory and others have shown that ovarian cancer cells in suspension have a propensity to aggregate and form non-proliferating cell clusters or spheroids [16, 44]. Further to this observation, we report here that cells in dormant EOC spheroids have reduced metabolic activity and induce the LKB1-AMPK metabolic stress response pathway. AMPK activity is enhanced in quiescent ovarian cancer spheroids, and in a reciprocal fashion, pharmacologic activation of AMPK in proliferating, adherent ovarian cancer cells leads to cytostasis. We have also uncovered a novel phenomenon where LKB1 is required for ovarian cancer cell survival and resistance to chemotherapy treatment in spheroids, whereas AMPK, the primary downstream substrate of LKB1, is dispensable. This implies that LKB1 utilizes an AMPK-independent signal to promote cell survival in metastatic ovarian cancer spheroid cells. In addition, ours is the first study to demonstrate the maintenance of LKB1 expression in ovarian cancer cells and its potential functional requirement during metastasis.

**Figure 6:**
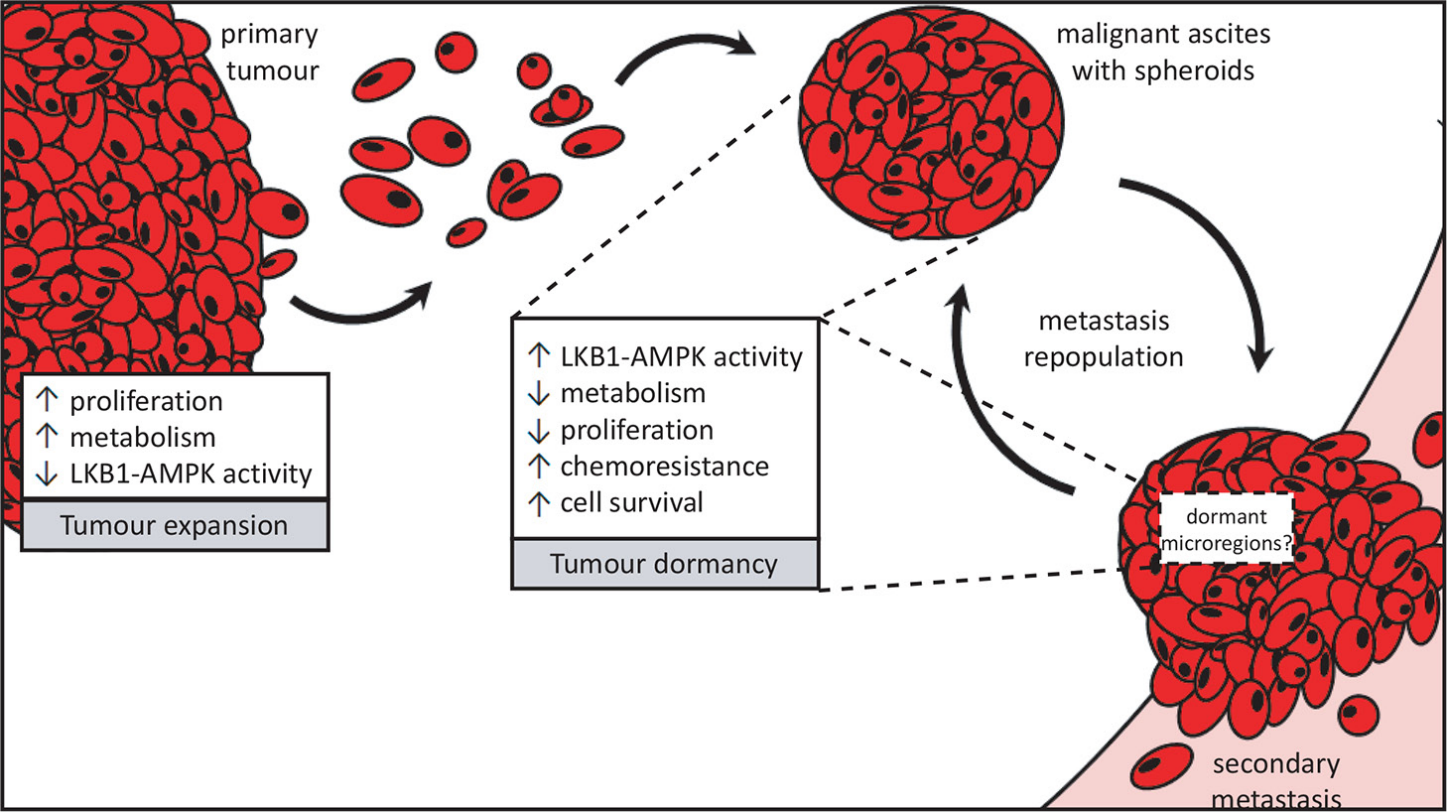
Proposed divergent roles for LKB1-AMPK signalling in metastatic ovarian cancer Ovarian cancer metastasis occurs via direct dissemination of primary tumour cells into the peritoneal cavity. Single cells in suspension will readily undergo detachment-induced apoptosis, or anoikis, but the formation into multicellular aggregates called spheroids protects them from cell death. Spheroids possess an enhanced ability to reattach onto the serosal surfaces of peritoneal organs upon which the ovarian tumour cells make a “dormant-to-proliferative switch” and grow to establish secondary metastases. Rapidly-expanding tumour growth (whether in the primary tumour or metastases) would require reduced LKB1-AMPK signalling to allow for increased cell proliferation and anabolic metabolism. Spheroids, on the other hand, adopt a number of pathobiological changes which we propose contribute to their dormant phenotype and facilitate cell survival during metastatic transit in suspension. Our new results support the role for active LKB1-AMPK signalling contributing to ovarian cancer cell dormancy in spheroids by reducing energy metabolism and cell proliferation, yet promote cell survival and carboplatin-resistance in EOC spheroids.

Expansive tumour growth is typically dependent on over-proliferative malignant cells that lack the normal response to induce protective growth arrest [45]. Under nutrient-rich conditions, proliferative cancer cells should have low or absent levels of active AMPK signalling. Indeed, we show that expression of phosphorylated AMPKα is marginal in the majority of adherent proliferating EOC cells. AMPK activity, however, is significantly elevated upon spheroid formation in line with the cellular quiescent phenotype of these structures [16]. AICAR and A-769662 both induce a potent cytostatic response in proliferating adherent cells without any significant cell death due to induction of apoptosis; and further activation of AMPK using these agents in spheroids has little impact on proliferation or viability. A recent report demonstrated that the growth-suppressive effects of AICAR are independent of AMPK in glioma cells [46]. Thus, the decrease in ovarian cancer cell proliferation observed in ovarian cancer cells treated with AICAR may occur via AMPK-independent mechanisms. However, a tumour suppressive-like activity for AMPK has been observed in ovarian cancer cells via the overexpression of the β1-subunit of AMPK [47]. Taken together, suppressed AMPK signalling is likely required to sustain active ovarian tumour growth (Figure 6), and supports a general idea that this pathway possesses classical tumour suppressor function under these conditions. Indeed, pharmacologic activation of AMPK signaling in the context of gynecologic cancer treatment has been discussed as a possible strategy for prolonging disease-free interval in patients [48, 49].

It is reasonable to postulate that LKB1 function may possess contrasting context-specific activities during steps of ovarian cancer progression (Figure 6). A recent report of a conditional mouse model for serous ovarian carcinoma determined that loss of *Stk11* in the context of *Pten* loss within the OSE leads to the development of high-grade papillary serous ovarian carcinomas [22]. In other cancer models, LKB1 loss-of-function has been shown to accelerate tumorigenesis in conjunction with p53 [50], *Kras* [51], and c-myc [52], as well. Although the study by Tanwar and colleagues [22] implicates loss of Lkb1 function in the initiation of ovarian cancer, genetic ablation was performed in the ovarian surface epithelium and not the secretory epithelium of oviduct; the secretory epithelium of the distal fallopian tube is now considered the site of origin for high-grade serous ovarian cancer [53, 54]. Also, we present TCGA data showing the frequency of homozygous loss of *STK11* is extremely low in high-grade serous ovarian tumours. Therefore, the significance of the results of the genetically-modified mouse model to human ovarian cancer is unclear. Recently, George and colleagues have reported reduced LKB1 protein expression in serous tubal intraepithelial carcinomas and high-grade serous ovarian tumours, again suggesting a tumour suppressor function [23]. In contrast, we clearly demonstrate that the majority of EOC solid tumour specimens, primary ascites-derived cells and established ovarian cancer cell lines retain detectable LKB1 protein expression since almost all serous ovarian cancers retain at least one *STK11* allele. Interestingly, we also identified copy number gains or amplifications in *PRKAA1*, encoding AMPKα1, suggesting that there may be compensatory mechanisms to upregulate AMPK activity in late-stage ovarian tumours harbouring reduced LKB1 in order to maintain a functional pathway for tumour cell survival during metastasis. Our data imply that although heterozygous loss of *STK11* with reduction in LKB1 expression may act to predispose to ovarian cancer initiation, maintenance of functional LKB1 signalling is likely essential during metastatic progression to promote cell survival in spheroids and fuel recurrence of chemo-resistant ovarian cancer (Figure 6).

LKB1-AMPK signalling represents an immediate response to metabolic stress and reduced energy supply to downregulate anabolic metabolism and shunt pathways to utilize alternative energy substrates [17, 55]. It has been shown in other cell systems that LKB1-AMPK signalling is an important mediator of protecting detached epithelial cells from anoikis [24, 25]. We show that targeted knockdown of *STK11* yielded a significant reduction in ovarian cancer spheroid cell viability, yet there was no effect when AMPKα1 expression was reduced. We confirmed that there was no compensatory effect of AMPKα2 expression in *PRKAA1*-knockdown spheroid cells that could explain this lack of effect, nor did *PRKAA2* siRNA elicit any change in total AMPKα expression in ovarian cancer cell lines (data not shown). This implies that LKB1 plays an important role in mediating anoikis-resistance in EOC spheroids, and perhaps other characteristics of the dormant phenotype of these structures, independent of AMPK.

AMPK is the most studied downstream target of LKB1. However, LKB1 has been called a ‘master kinase’ given its ability to phosphorylate at least 12 other downstream proteins, referred to as AMPK-related kinases (ARKs) [56]. Perhaps one or more of these ARKs are critical downstream mediators of the LKB1-dependent effects on cell viability in ovarian cancer spheroids. For example, salt inducible kinase 2 (SIK2) is overexpressed in high-grade serous ovarian cancer and has been shown to function in cell division through its regulation of mitotic spindle formation by phosphorylating proteins of the centrosome [57]. Likewise, SIK3 has been documented as a putative tumour-associated antigen with expression in 55% of ovarian cancer samples, and, like SIK2, can control cell cycle progression at the G1/S checkpoint [58]. Whether these ARKs represent AMPK-independent targets of LKB1 signalling in EOC spheroids is unknown; as such, further studies are needed to determine which of the numerous substrates downstream from LKB1 are mediating its effects on maintaining cell viability in ovarian cancer spheroids.

EOC spheroids have the capacity to harbour a niche of chemotherapy-resistant cells. Our data supports this idea, and we provide the first evidence that LKB1-AMPK signalling may play a significant role in adaptive resistance mechanisms in these metastasis-promoting structures. Phosphorylation of both LKB1 and AMPKα are increased in spheroids and act to support a dormant non-proliferative state, which would thereby decrease the efficacy of standard chemotherapeutic agents. In fact, carboplatin and paclitaxel treatment of EOC cells increase phosphorylated AMPKα levels (data not shown), and targeted knockdown of its upstream kinase LKB1 renders EOC spheroids re-sensitized to carboplatin-induced cell death. This implies that a combination of chemotherapy with novel agents eliciting LKB1-AMPK pathway inhibition would be efficacious as an upfront approach to treat ovarian cancer patients with late-stage disease to better eradicate residual dormant micro-metastases and reduce chemo-resistant disease recurrence.

The general concept of tumour dormancy in cancer research, though provocative, is far from proven using experimental models. Direct empirical dissection of tumour dormancy mechanisms *in vivo* would be quite challenging; thus, we claim that using spheroids as a tractable model system to perform extensive *in vitro* investigations will facilitate the discovery of potential tumour dormancy mechanisms. These results could then be applied to complementary and direct *in vivo* tumour models and patient-derived tumour samples.

## MATERIALS AND METHODS

### Cell culture

SkOV3 cells were cultured using DMEM (Wisent, St. Bruno, Canada) with 5% fetal bovine serum (FBS; Wisent). Hey, HeyA8 cells, and HeyC2 cells (gift from G. Mills, MD Anderson) were cultured using RPMI-1640 (Wisent) with 5% FBS. OVCAR3, OVCAR5, and OVCAR8 (purchased from ATCC, Manassas, VA) were cultured using RPMI-1640 with 5% FBS. CaOV3 cells (ATCC) were cultured using DMEM with 10% FBS. OW-7 and 105-C cell lines (gift from H. Hirte, McMaster University) were grown in RPMI-1640 with 10% FBS. Ascites fluid was collected from patients, the majority of whom were diagnosed with advanced-stage, high-grade serous EOC (Supplementary Table S1), and used to establish primary cell cultures as previously described [59]. Cell lines iOvCa142 and iOvCa147-E2 were generated from ascites samples EOC142 and EOC147 collected at our centre.

For the majority of experiments assayed using spheroids, cells were seeded to 24-well ultra-low attachment (ULA) cluster plates (Corning) at a density of 5 × 10^4^ cells per well. For protein isolation from spheroid cells, cells were seeded at 5 × 10^5^ cells per well of a 6-well ULA cluster plate. Cells were cultured for 3 d in suspension to form spheroids for all experiments, unless otherwise specified. Native spheroids were isolated directly from ascites fluid by filtration through a 40 μm cell strainer (Becton Dickinson), washed with phosphate-buffered saline (PBS) into a collection tube with protein lysis buffer for immunoblot or embedded in OCT to prepare frozen sections for immunofluorescence. To compare native spheroid samples with matched adherent cells, cells at passage-0 were used. All work with patient materials has been approved by The University of Western Ontario Health Sciences Research Ethics Board (Protocol # 12668E and 16391E).

### TCGA and CCLE analysis

Datasets from The Cancer Genome Atlas analysis of ovarian serous cystadenocarcinoma samples were downloaded from the University of California Santa Cruz Cancer Genomics Browser (https://genome-cancer.ucsc.edu) [60] and from the Memorial Sloan-Kettering Cancer Center's cBioPortal for Cancer Genomics (http://www.cbioportal.org/) [31, 32]. Raw data was analyzed using the GISTIC2 method to generate gene-level copy-number variation (CNV) estimates and downloaded as either thresholded copy-number calls or as log2-transformed CNV values. Reverse-phase protein array data was downloaded as either natural log-transformed values or z-scores. Copy number and mRNA expression data on ovarian cancer cell lines of the Cancer Cell Line Encyclopedia [61] were accessed from cBioPortal.

### Immunoblotting and immunofluorescence

Protein lysates were generated from cells in adherent and spheroid culture as previously described [62]. Protein lysates from solid tumour specimens were prepared by homogenizing flash-frozen tissue in lysis buffer [50 mM HEPES pH7.4, 150 mM NaCl, 10% glycerol, 1.5 mM MgCl_2_, 1 mM EGTA, 1 mM sodium orthovanadate, 10 mM sodium pyrophosphate, 10 mM NaF, 1% Triton X-100, 1% sodium deoxycholate, 0.1% SDS, 1 mM PMSF, 1X protease inhibitor cocktail (Roche, Laval, Quebec, Canada)]. Antibodies used to detect p-AMPKα Thr172 (#2535), AMPKα (#5832), p-LKB1 Ser428 (#3482), LKB1 (#3050), p-p70S6K1 Thr389 (#9234), p-ACC (#3661), ACC (#3676) and p70S6K1 (#2708) expression by immunoblotting were obtained from Cell Signaling Technology (Danvers, MA). Anti-Tubulin antibody was obtained from Sigma (Mississauga, ON, Canada). AICAR was purchased from Caymen Chemical Company (Ann Arbor, MI) and A-769662 from Tocris Bioscience (Bristol, UK). Immunofluorescence analysis was performed on cryosections of native spheroids that were fixed (4% formaldehyde), permeabilized (0.1% Triton X-100 in PBS), and blocked (5% BSA in 0.1% Triton X-100) before incubation with p-AMPKα antibody (#ab51110) from Abcam^®^ Inc. (Cambridge, MA). Following primary antibody incubation and PBS washes, sections were incubated for 1 hour with anti-rabbit FITC secondary antibody (1:250 dilution; Sigma-Aldrich) and 4′, 6-diamidino-2-phenylindole (DAPI; 1:1000) and mounted with Vectashield (Vector Laboratories, Burlingame CA, USA). Fluorescence images were captured using an Olympus AX70 upright microscope and ImagePro image capture software.

### Cell viability and ATP assays

Cells were seeded to either 24-well tissue culture plastic or ultra-low attachment (ULA) plates at a density of 1 × 10^4^ per well to form adherent cultures or 5 × 10^4^ per well to form spheroids, respectively. Treatment was initiated at time of seeding for cells in suspension while cells under adherent conditions were given 12 h to adhere before commencing treatment. At 72 h post-treatment, spheroids were collected, pelleted and left in media (100 μL), at which point CellTiter-Glo^®^ reagent (Promega, Madison, WI) was added (1:1 v/v ratio). Under adherent conditions, cells were harvested directly in CellTiter-Glo^®^ after 20 min incubation. All samples were subjected to a freeze/thaw cycle prior to analysis. Approximately 200 μL of the mixture was added to a white-walled 96-well microplate and luminescence signal was detected using a Wallac 1420 Victor 2 spectrophotometer (Perkin-Elmer, Waltham, MA) and normalized to vehicle-treated cells. For measuring cell viability after carboplatin treatment, adherent cells and spheroids were treated with 50 μM carboplatin (LHSC Pharmacy, London, ON) and assays were performed after 72 h as described above.

### Clonogenic assays

Cells were seeded at 1 000 cells per well of a 6-well dish and treated with 1 mM AICAR or 100 μM A-769662 for 72 h. Subsequently, medium was replaced with complete growth medium without AMPK agonists until detectable colonies were observed. Cells were stained with Hema-3 and the total number of colonies was counted in each well.

### Flow cytometry

Cells treated with were pulse-labeled with 10 μM bromodeoxyuridine (BrdU; GE Healthcare, Buckinghamshire, UK) for 2 h. Cells were fixed in 95% ethanol and stored at 4°C. Cells were stained in the following solutions: 2N HCl/0.5% TritonX-100, 0.1M NaB_4_O_4_ pH 8.5, mouse anti-BrdU primary antibody (1:50; Becton Dickinson), anti-Mouse FITC-conjugated secondary antibody (1:250; Vector Laboratories, Burlingame, CA), and PI Staining Solution [PBS with 2% FBS, 0.25 μg/μL RNase, 10 μg/mL PI]. Samples were incubated at 37°C for 30 min, then overnight at 4°C. The following day, labeled cells were filtered using 40 μm cell-strainers and flow cytometry using a Beckman Coulter Epics XL-MCL (10 000 events/replicate, three replicates/experiment).

### siRNA transfections

Cells were plated at a density of approximately 1 × 10^5^ cells per 35 mm well in antibiotic-free media, and transfections were performed the next day as per manufacturer's protocol (Dharmacon; Thermo Fisher Scientific Inc., Waltham, MA). DharmaFECT1 was used for all cell lines and primary EOC cells, except DharmaFECT3 was used for SkOV3 cells. Briefly, 1 μL of DharmaFECT1 or 4 μL of DharmaFECT3 was combined with 10 nM siRNA in 1 mL of media and incubated for 20 min; the siRNA/DharmaFECT complexes were then added directly to each well. Media was removed 24 h following transfection and replaced with antibiotic-free growth media. At this point, the cells were incubated approximately 72 h following transfection. Trypsinized cells were counted directly using a TC10 cell counter (Biorad) to determine adherent cell viability, then seeded at 5 × 10^4^ cells into 24-well ULA cluster plates; cell viability was determined at 72 h post-seeding using CellTiter-Glo^®^. *PRKAA1* (M-005027-02) and *STK11* (M-005035-02) siGENOME SMARTpool siRNAs were obtained from Dharmacon (Thermo Fisher Scientific Inc., Waltham, MA). Western blot analysis was performed on adherent cell lysates at 72 h post-transfection and 72 h post-seeding to ULA plates for spheroid lysates.

### Graphing and statistical analysis

All graphs were generated and statistical analyses were performed using GraphPad Prism 5 (GraphPad Software, San Diego, CA). Data were expressed as mean ± SEM, and statistical analyses were either Student's *t*-test or Analysis of Variance with Tukey's Multiple Comparison Test. Tests of significance were set at *p* < 0.05.

## SUPPLEMENTARY FIGURES AND TABLE


