# Supplementary material for: Intact LKB1 activity is required for survival of dormant ovarian cancer spheroids
Source: Oncotarget. 2015 Jun 5;6(26):22424–38. doi: 10.18632/oncotarget.4211 (PMC4673173; doi:10.18632/oncotarget.4211)
Supplement: Supplementary file 1 [file oncotarget-06-22424-s001.pdf]

SUPPLEMENTARY FIGURES AND TABLE

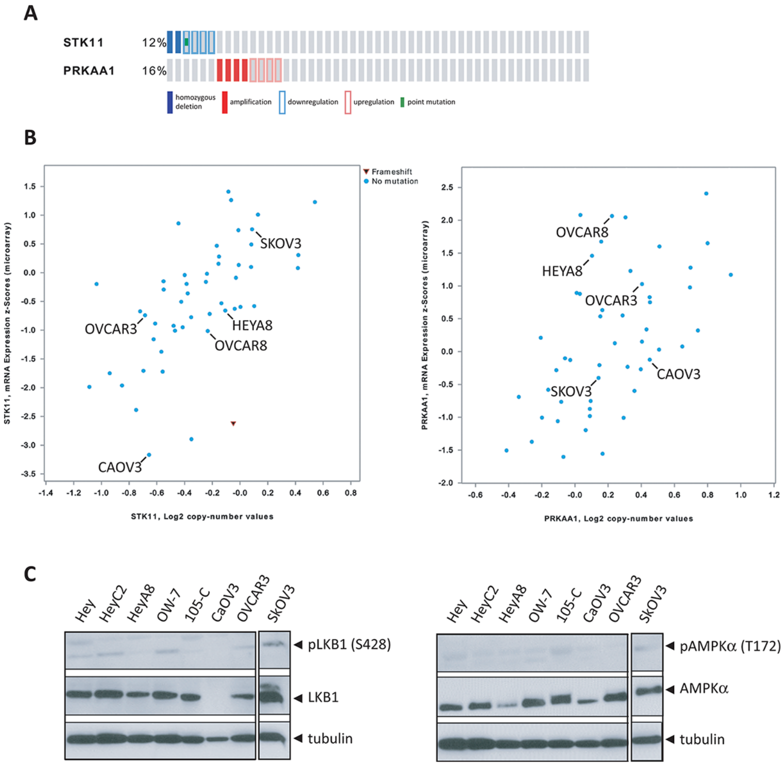

Supplementary Figure S1: Status of LKB1 (*STK11*) and AMPKα (*PRKAA1*) in ovarian cancer cell lines. A. Oncoprint analysis of copy number at the *STK11* and *PRKAA1* gene loci are depicted for 51 ovarian cancer cell lines obtained using the Cancer Cell Line Encyclopedia (CCLE) dataset from cBioPortal. Homozygous deletion (solid blue), amplification (solid red), downregulated expression (blue outline), upregulated expression (red outline), and point mutation (small green box) are shown. **B.** Scatterplot of mRNA expression z-scores against $\log_2$-transformed copy-number values using CCLE data from cBioPortal. Cell lines used in the present study are indicated. **C.** Immunoblot analysis of p-LKB1 (Ser428), LKB1, p-AMPKα (Thr172) and AMPKα was performed on proliferating ovarian cancer cell lines cultured under adherent conditions. Tubulin served as a control for protein loading.

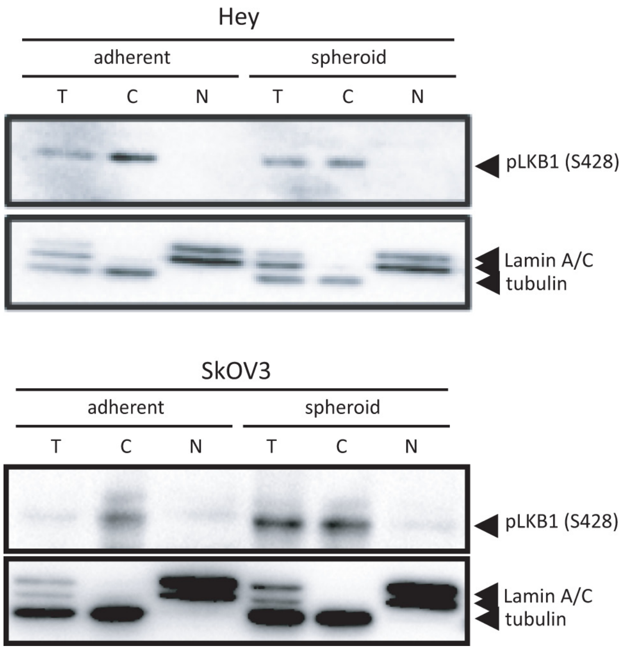

Supplementary Figure S2: LKB1 is localized to the cytoplasm in adherent and spheroid EOC cells. Immunoblot analysis of LKB1 on whole-cell (T), cytoplasmic (C), and nuclear (N) protein extracts isolated from Hey and SkOV3 ovarian cancer cell lines to determine subcellular localization. Lamin A/C and tubulin were used as controls for nucleus and cytoplasm, respectively.

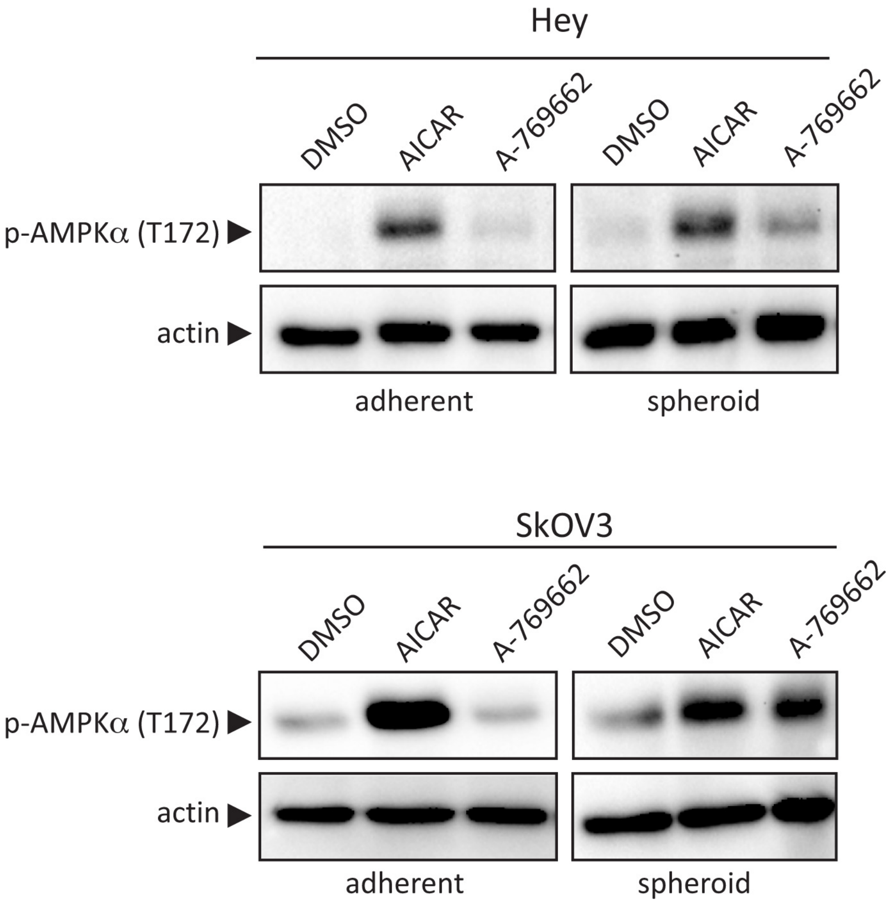

Supplementary Figure S3: Verification of AMPK activation of ovarian cancer cells treated in adherent and spheroid culture. Immunoblot analysis of p-AMPKα (Thr172) expression in Hey and SkOV3 cells grown in adherent or spheroid culture and treated with 1 mM AICAR or 100 µM A-769662 for 24 h; DMSO was used as vehicle control. Actin was used to control for protein loading.

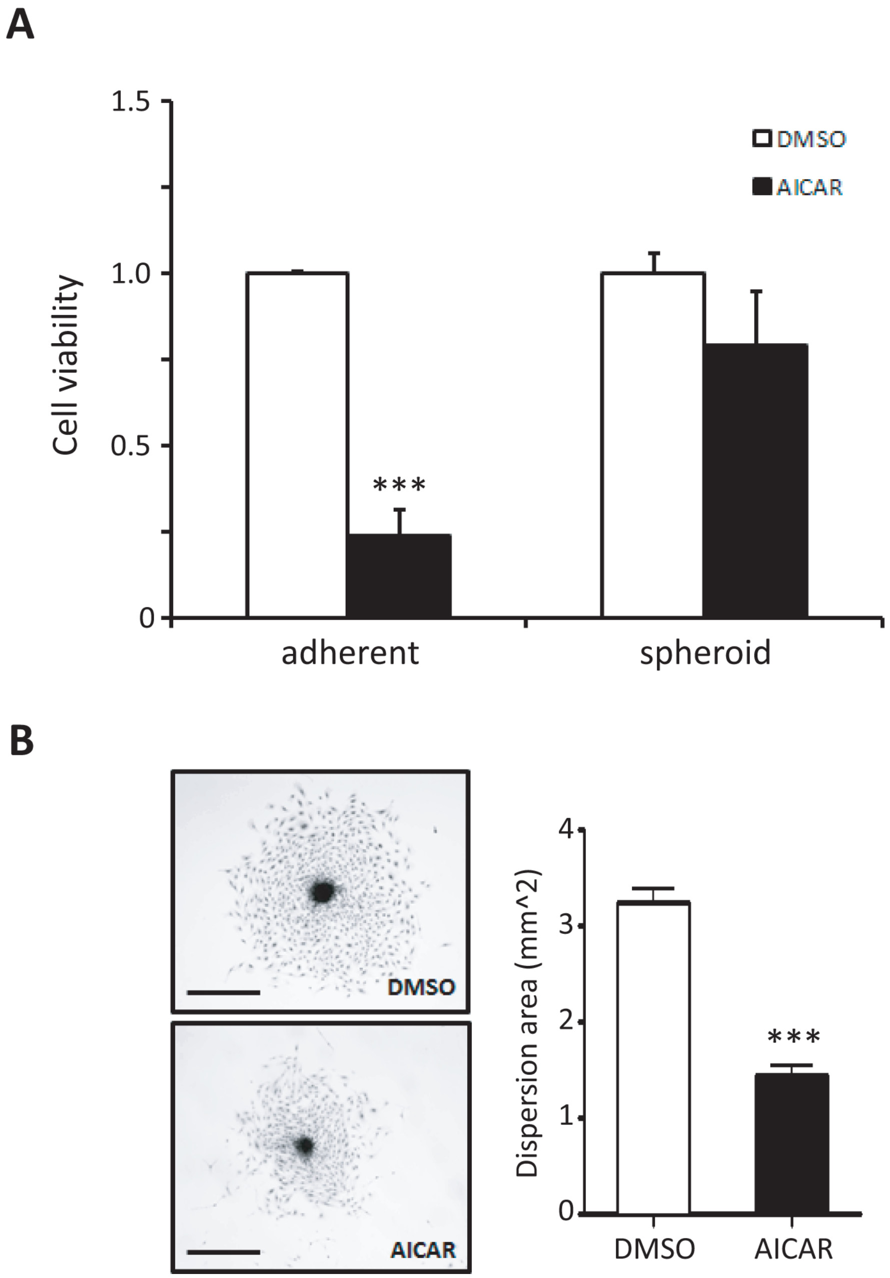

Supplementary Figure S4: AICAR treatment of primary EOC reduces cell proliferation. A. Cell viability was determined for AICAR-treated primary cultures of ascites-derived cells collected from ovarian cancer patients. Cells were treated with 1 mM AICAR or DMSO vehicle control for 72 h in adherent culture (n = 7) or as spheroids in suspension (n = 9) (***, $p < 0.001$). **B.** Spheroids formed from ascites-derived cells (n = 9) and treated with 1 mM AICAR for 72 h were seeded for reattachment to tissue culture plastic. Area of cell dispersion was quantified at 72 h post-reattachment using ImageJ and Student's t-test for statistical significance (***, $p < 0.001$). Representative image of one ascites-derived spheroid sample (EOC154) is depicted. Scale bar: 1 mm.

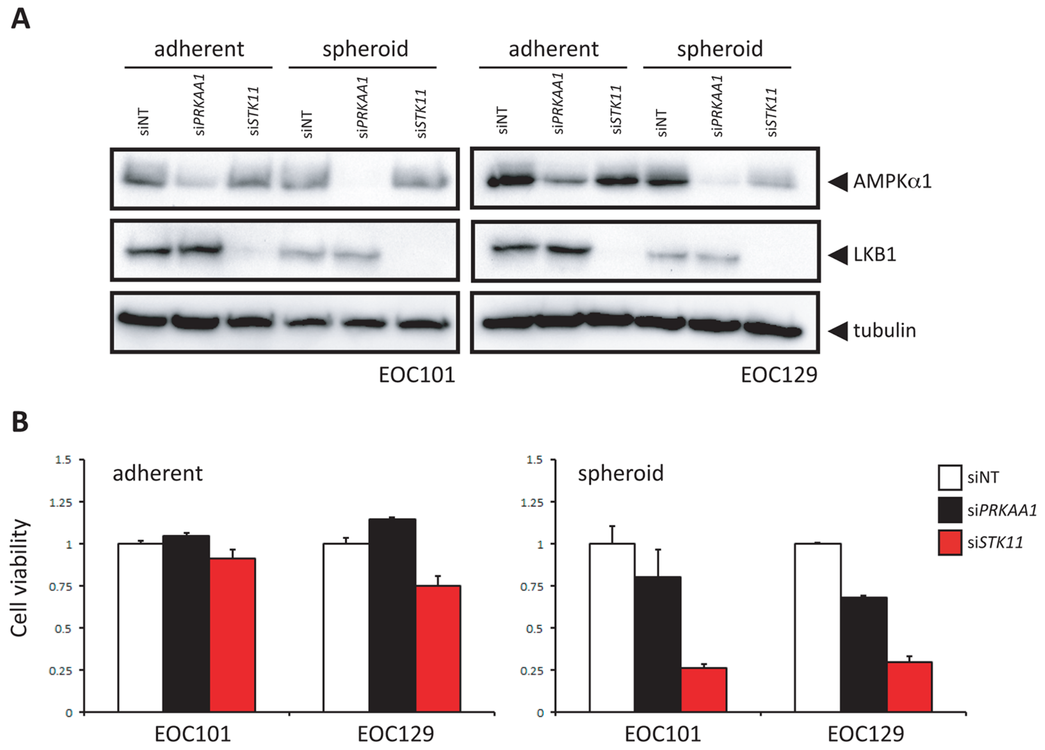

Supplementary Figure S5: *STK11* knockdown in primary ascites-derived EOC cells reduces spheroid cell viability. A. Immunoblot analysis of LKB1 and AMPKα expression in two ascites-derived primary human EOC samples transfected with *STK11* and *PRKAA1* siRNA pools, or control siRNA (siNT). Expression was determined 72 h after transfection of adherent cells and 72 h after spheroid formation. **B.** Cell viability was determined after 72 h using CellTiter-Glo® assay on siRNA-transfected primary EOC cells cultured as adherent cells or spheroids.

Supplementary Table S1: Summary of clinical data for ovarian cancer patient samples used in this study

Sample	Age	Histological Subtype	Grade	Stage
EOC57	46	Serous	High	IIIC
EOC61	78	Serous	High	IIIC
EOC87	47	Serous	High	IV
EOC98	51	Serous	High	IIIC
EOC101	43	Serous	High	IIIC
EOC105[a]	43	Serous (70%)/clear cell (30%)	High	IIC
EOC110	43	Serous	High	IIIC
EOC116	64	Serous	High	IIIC
EOC122	56	Serous	High	IIIC
EOC129	74	Serous	High	IIIC
EOC130	59	Serous	High	IIIC
EOC132	59	Serous	High	IIIC
EOC136	42	Serous	High	IV
EOC137	77	Serous	High	IIIC
EOC140	76	Poorly differentiated	High	IIIC
EOC148	67	Serous	High	IC[b]
EOC149	69	Serous	High	IIIC
EOC153	48	Serous (60%)/ endometrioid (40%)	High	IIA
EOC154	66	Serous	High	IIIC
EOC155	66	Serous	High	IIIC
EOC156	67	Serous	High	IIIC
EOC158	45	Serous	High	IIIC
EOC159	57	Serous	High	n.a[c]
EOC161	67	Serous	High	IIIC
EOC166	64	Serous	High	IIIC
EOC170	77	Serous (70%)/clear cell (30%)	High	IIIC
ECO171	65	Serous	High	IIIC
iOvCa142[a]	44	Serous (70%)/clear cell (30%)	High	IIC
iOvCa147-E2[a]	44	Serous (70%)/clear cell (30%)	High	IIC

[a]- EOC105, EOC142 and EOC147 are three independent ascites samples collected from the same patient separated by 14 months. The patient received 9 cycles of carboplatin/paclitaxel initiated after EOC105 sample was collected.
[b]- Stage was defined as at least IC for this patient.
[c]- n.a., not available
